# Successful treatment of atrial flutter post-radiofrequency ablation for atrial fibrillation following atrial septal defect occlusion: a case report of pulsed field ablation

**DOI:** 10.1093/ehjcr/ytae558

**Published:** 2024-10-22

**Authors:** Jing Hu, Ligang Ding, Evan Gunawan, Hengli Lai, Yan Yao

**Affiliations:** Department of Cardiovascular, Jiangxi Provincial People’s Hospital, The First Affiliated Hospital of Nanchang Medical College, Nanchang, Jiangxi, China; Cardiac Arrhythmia Center, Fuwai Hospital, National Center for Cardiovascular Diseases, Chinese Academy of Medical Sciences and Peking Union Medical College, Beijing, China; Binawaluya Hospital Cardiac Centre, Jakarta, Indonesia; Department of Cardiovascular, Jiangxi Provincial People’s Hospital, The First Affiliated Hospital of Nanchang Medical College, Nanchang, Jiangxi, China; Cardiac Arrhythmia Center, Fuwai Hospital, National Center for Cardiovascular Diseases, Chinese Academy of Medical Sciences and Peking Union Medical College, Beijing, China

**Keywords:** Pulsed field ablation, Atrial flutter, Radiofrequency ablation, Atrial septal defect occlusion, Case report

## Abstract

**Background:**

Atrial flutter (AFL) is a common arrhythmia following radiofrequency ablation (RFA) for atrial fibrillation (AF), with varying incidence depending on the ablation strategy. Patients with prior atrial septal defect (ASD) occlusion pose challenges for ablation, particularly when the lesions are located near the occluder. Pulsed field ablation (PFA) has emerged as a promising alternative to RFA for the treatment of AF or AFL; however, its use in patients with ASD occlusion remains unexplored.

**Case summary:**

We present the case of a 46-year-old female with a history of ASD occlusion and subsequent RFA for AF. Despite the initial success, she developed symptomatic AFL 3 months post-procedure. Intracardiac echocardiography (ICE)–guided transseptal puncture guided by ICE revealed an AFL originating from the slow conduction area around the ASD occluder. Pulsed field ablation was successfully performed, and AFL was terminated without complications. Post-procedural follow-up demonstrated maintenance of sinus rhythm.

**Discussion:**

Patients with ASD occlusion present unique challenges for ablation, including difficulties in transseptal puncture and risk of injury to the occluder. Pulsed field ablation offers a potential solution, with studies showing fewer reconnected pulmonary veins and larger lesion creation compared with traditional methods. In our case, PFA effectively terminated the refractory AFL, highlighting its utility in this patient population. Moreover, the use of the Jinjiang PFA catheter with pulse circuit self-checking technology ensured procedural safety, particularly near the occluder.

**Learning points**

1. Intracardiac ultrasound guidance is safe and feasible for difficult atrial septal puncture after closure of atrial septal defect.
2. The pulsed field ablation represents a safe and effective therapeutic option for the treatment of atrial flutter refractory to radiofrequency ablation, use of the Jinjiang PFA catheter with pulse circuit self-checking technology ensured procedural safety, particularly for the ablation of the lesions around the occluder.

## Introduction

Atrial flutter (AFL) is a common occurrence in some patients after atrial fibrillation (AF) ablation, with an incidence ranging from <5% to 40%, depending on the ablation strategy and scope.^1^ Ablation procedures pose challenges in individuals with atrial septal defect (ASD) occluders and a history of radiofrequency ablation (RFA) for AF, particularly when the lesions encroach around the occluder. In recent years, pulsed field ablation (PFA) has become one of the treatment options for atrial arrhythmia.^2–4^ At the time of repeat ablation, patients showed a significantly lower number of reconnected pulmonary veins than those initially treated with cryoablation or RFA. Moreover, the PFA catheter can create a larger lesion that affects the critical isthmus, which is not achieved by RFA.^5^ Herein, we present a case of successful PFA for AFL refractory to RFA in a patient with a history of ASD occlusion, highlighting the potential utility of PFA in this challenging patient population.

## Summary figure

**Figure:**
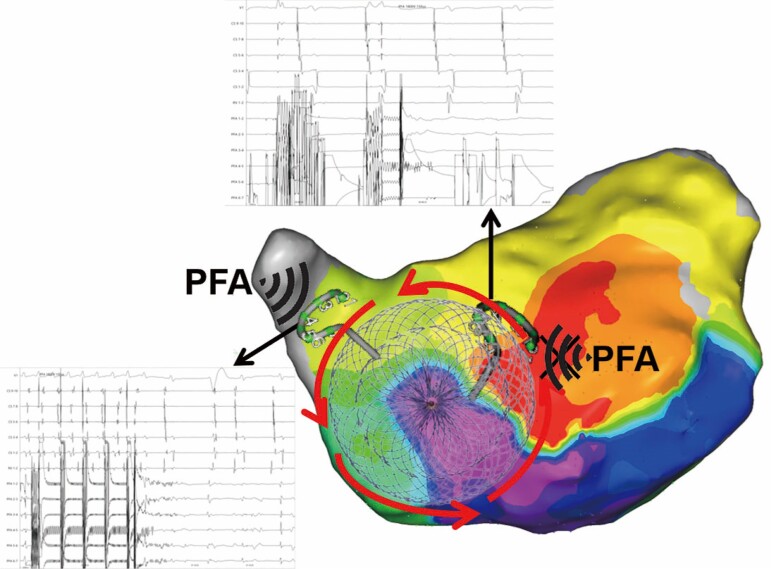


## Case report

**Figure 1:**
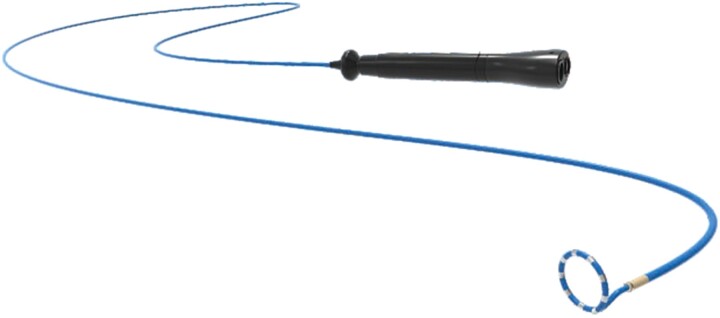
A circular-shaped pulsed field ablation catheter, can mapping, model reconstruction, and ablation. The distal end was 5.5 Fr and the proximal end was 8 Fr in diameter. The diameter of circular catheter is 15 mm.

**Figure 2:**
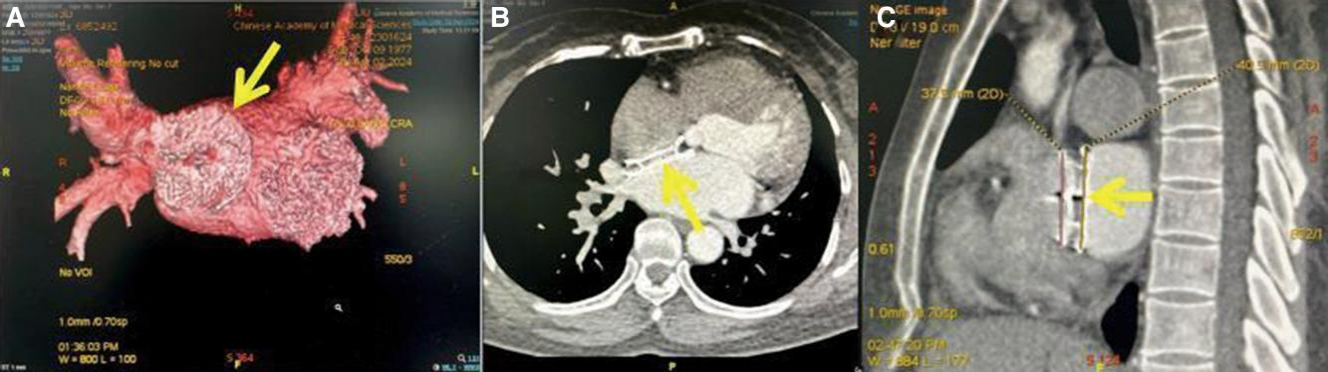
The arrows indicate a huge atrial septum occluder in the left atrial cardiac computed tomography: 3D reconstructions (*A*), transverse sections (*B*), and sagittal sections (*C*).

A 46-year-old female with a history of ASD occlusion [XJFS26 (Lifetech Scientific, Inc., China)] presented with persistent symptomatic AF refractory to medical management. She underwent RFA with pulmonary vein isolation (PVI) and additional ablation lines (left atrial roof line, left atrial anterior wall to the right superior pulmonary vein (PV), and right atrial middle of the crista terminalis to the inferior vena cava). However, 3 months post-procedure, the patient experienced recurrent palpitations. Electrocardiography (ECG) revealed an AFL. Despite therapy with antiarrhythmic medications, including atenolol and propafenone, which were stopped 2 weeks before admission, the patient still had recurrent palpitations, prompting consideration for repeat ablation. Physical examination and laboratory tests on admission did not reveal significant abnormalities. Given the patient’s complex cardiac anatomy and prior ASD occlusion, LEAD-PFA [Sichuan Jinjiang Electronic Medical Device Technology Co., Ltd. (JJET)], a proprietary bipolar PFA system, was chosen as the preferred ablation modality. The system was designed for PVI and utilized a circular PFA catheter [PulsedFA (JJET)] (*Figure 1*) embedded with three magnetic sensors, allowing model reconstruction, mapping, and ablation in one map with three-dimensional (3D) navigation (JJET).^6^ Pre-procedural imaging with cardiac computed tomography was performed to assess the atrial anatomy and exclude a thrombus, revealing the presence of a large spacer occluder (*Figure 2*).

**Figure 3:**
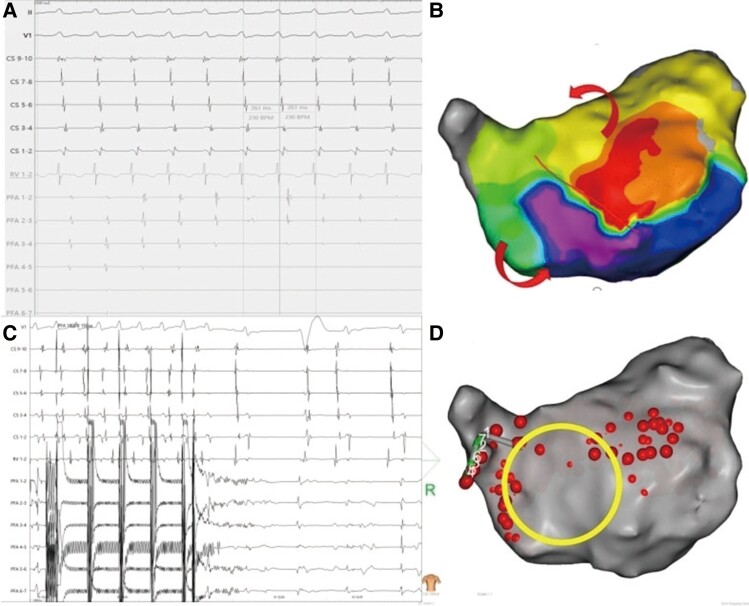
Mapping and ablation of first-type atrial flutter. (*A*) Electrocardiogram and intracardiac electrograms showing first-type atrial flutter. (*B*) Activation mapping reveals re-entry of the anterior wall, septum, and right superior pulmonary vein around the occluder; the red arrow indicates the re-entry circle. (*C* and *D*) Electrocardiogram and intracardiac electrogram recordings during pulsed field ablation with termination of the first-type atrial flutter (*C*) and catheter position (*D*); the big circle indicates the atrial defect occluder.

**Figure 4:**
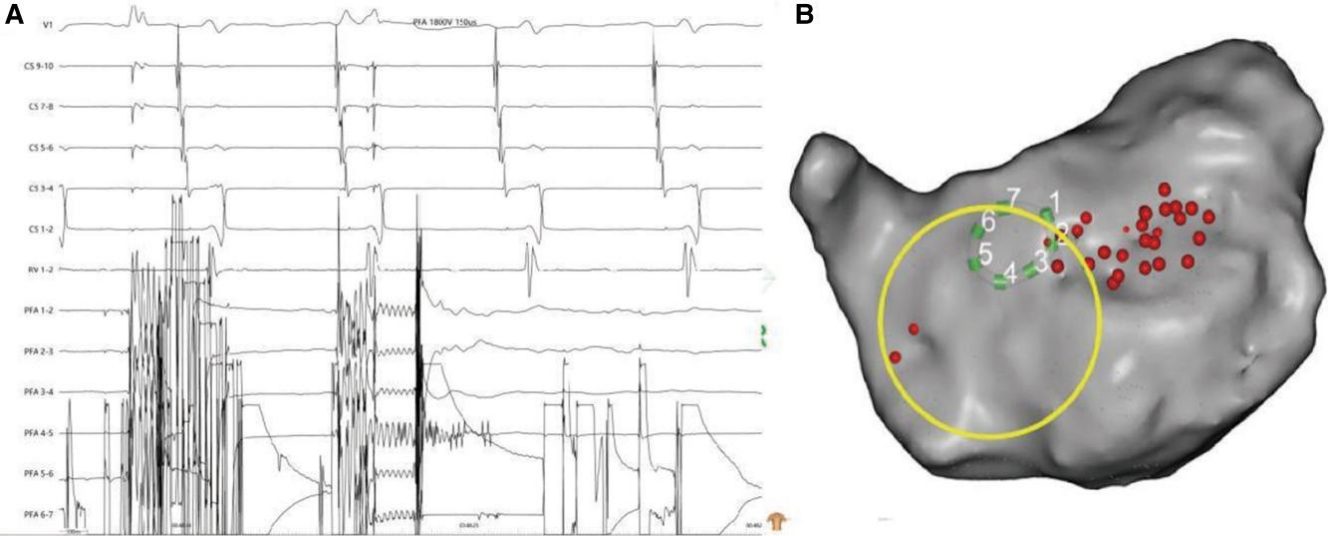
Electrocardiogram and intracardiac electrograms recording the interference (*A*) around the occluder (*B*); the impedance was too low to discharge, and the big circle indicated the atrial defect occluder.

**Figure 5:**
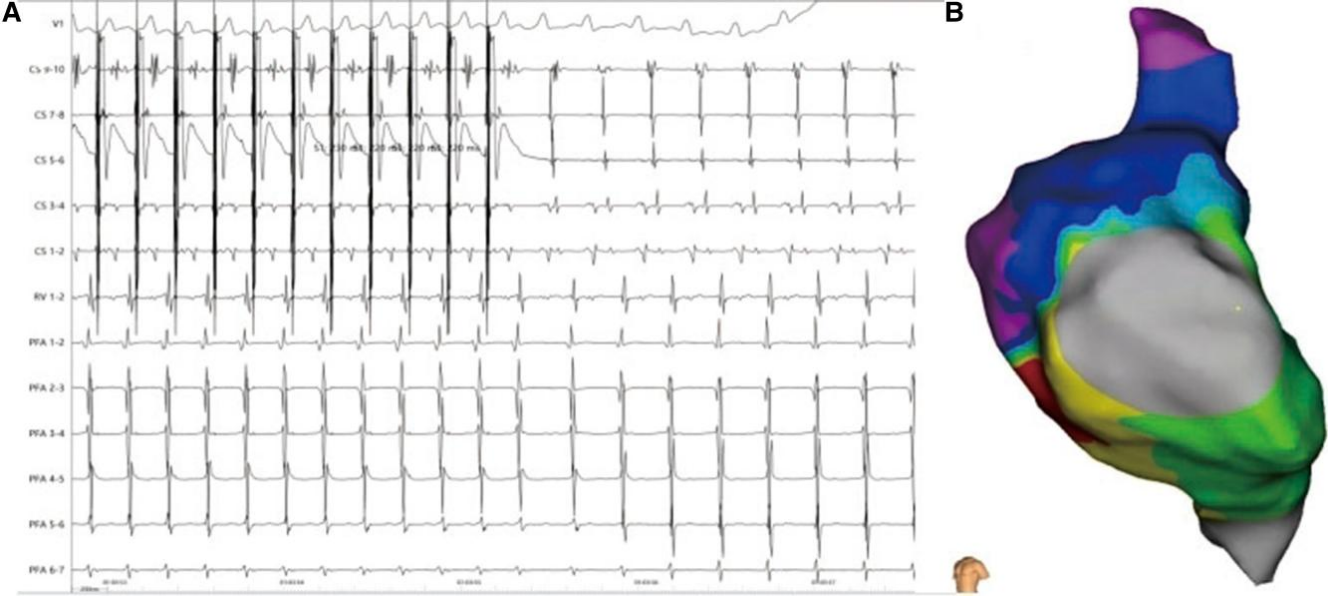
(*A*) Electrocardiogram and intracardiac electrograms recording the second-type atrial flutter. (*B*) Activation mapping reveals the re-entry of the tricuspid valve.

First, femoral vein puncture was performed under local anaesthesia, and a coronary sinus (CS) electrode was inserted. Electrophysiological (EPS) examination revealed the first type of AFL, with the earliest activation observed at CS 9–10 and a total cycle length (TCL) of 261 ms (*Figure 3A*). Entrainment mapping suggested involvement of the left atrium. Subsequently, transseptal puncture was performed under intracardiac echocardiographic (ICE) guidance (see Supplementary material online, *Videos S1*–*S3*). A 3D mapping system was employed to construct a left atrial model and reveal low-voltage areas in the right anterior and septal regions of the left atrium. Activation mapping indicated the main re-entry involving the anterior wall, septum, and right superior PV (*Figure 3B*; Supplementary material online, *Video S4*). Pulsed field ablation (voltage amplitude, 1800 V; intergroup period, 400 ms; effective time, 150 μs) was conducted at the anterior wall line and left atrial roof line, avoiding the ASD occluder, and ablation of the right superior PV terminated the AFL, restoring sinus rhythm (*Figure 3C* and *D*). During further ablation of the anterior wall line perimeter, electrode interference occurred at the marked position on the anterior wall (within the big circle) and frequent discharges failed because the impedance was too low (*Figure 4*). Subsequent EPS examination induced the second type of AFL, with CS 9–10 showing the earliest activation and a TCL of 256 ms (*Figure 5A*). Activation mapping suggested a counterclockwise re-entry involving the isthmus of the tricuspid valve (*Figure 5B*; Supplementary material online, *Video S5*). Pulsed field ablation at the isthmus of the tricuspid valve terminated AFL and restored sinus rhythm. Bidirectional block at the isthmus of the tricuspid valve was confirmed. Subsequent EPS examination did not induce tachycardia, and ablation was successfully completed.

No complications occurred during this procedure. Post-procedural ICE did not show any evidence of pericardial effusion. Sotalol (40 mg b.i.d.) was taken postoperatively and discontinued after 2 weeks. At the 3-month follow-up, the patient’s ECG revealed maintenance of sinus rhythm without any evidence of AFL recurrence.

## Discussion

To the best of our knowledge, this is the first successful use of PFA for the treatment of RFA-refractory AFL in a patient with a history of ASD occlusion. There are two difficulties for patients with left AFL after AF ablation following ASD occlusion: atrial septal puncture and safety of the ablation peri-occluder.

Access to the left atrium via transseptal puncture (single or double access) is a critical step in percutaneous catheter ablation of the AF or left AFL. Transseptal puncture can be challenging in the setting of a prior percutaneous ASD/patent foramen ovale occluder owing to an oversized device or alteration of septal anatomy, with limited or no visualization of the interatrial septum.^7^ In this case, a transseptal puncture was performed under ICE and fluoroscopic guidance, and the atrial septum was successfully punctured through the native septum of the anteroinferior edge of the occluder.

Gardziejczyk *et al*.^8^ found that PFA can be associated with more consistent scar formation, which can be crucial for difficult atrial tachycardia ablation. There are preclinical reports of better energy penetration through scars and fatty tissue compared with RF energy. Audiat *et al*.^9^ explore the challenges posed by an occluder during PFA of the left superior PV. This study found that an occluder can cause interference during the procedure, leading to issues such as electrical arcing and temperature increase. This interference can affect the effectiveness and safety of PFA by altering the electric field distribution and potentially damaging adjacent tissues. This case was a complex arrhythmia after ASD occlusion with recurrent AFL following RFA of atrial fibrillation. The lesion was located near the atrial septal occluder using LEAD-PFA successful ablation. Moreover, the LEAD-PFA impedance self-test function can load detection signals with frequencies ranging from 2000 to 200 000 Hz, convert the collected electrical signals into impedance signals through filtering, and obtain the current impedance change rate of the entire ablation circuit, which can prevent discharge when the impedance is too low or too high. For patients with atrial septal occluders, effective prevention of intraoperative metal collisions leads to short-circuit discharges between the electrodes, ensuring procedural safety.

## Conclusion

Pulsed field ablation is a safe and effective therapeutic option for the treatment of RFA-refractory AFL and ensures procedural safety, particularly for ablation of lesions around the occluder.

## Lead author biography


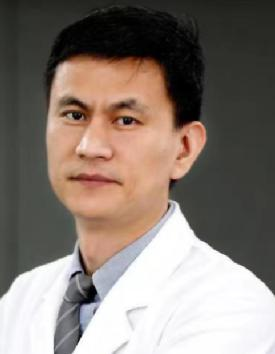
As a chief physician and doctor, his current practice focuses on complex catheter ablation, with a research focus on the application of pulsed field ablation on atrial and ventricular arrhythmias (translational and clinical research).

## Supplementary Material

ytae558_Supplementary_Data

## Data Availability

The data that support the findings of this study are available from the corresponding authors, upon reasonable request.

## Supplementary material

**Consent:** The authors confirm that written consent for submission and publication of this case report including image(s) and associated text has been obtained from the patient in line with the COPE guidelines.

**Funding:** This study was supported by the National High Level Hospital Clinical Research 350 Funding (2023-GSP-GG-22).
