# Supplementary material for: Successful treatment of atrial flutter post-radiofrequency ablation for atrial fibrillation following atrial septal defect occlusion: a case report of pulsed field ablation
Source: Eur Heart J Case Rep. 2024 Oct 22;8(11):ytae558. doi: 10.1093/ehjcr/ytae558 (PMC11558455; doi:10.1093/ehjcr/ytae558)
Supplement: ytae558_Supplementary_Data [file ytae558_supplementary_data.zip › Supplemental files.docx]

# Supplemental files


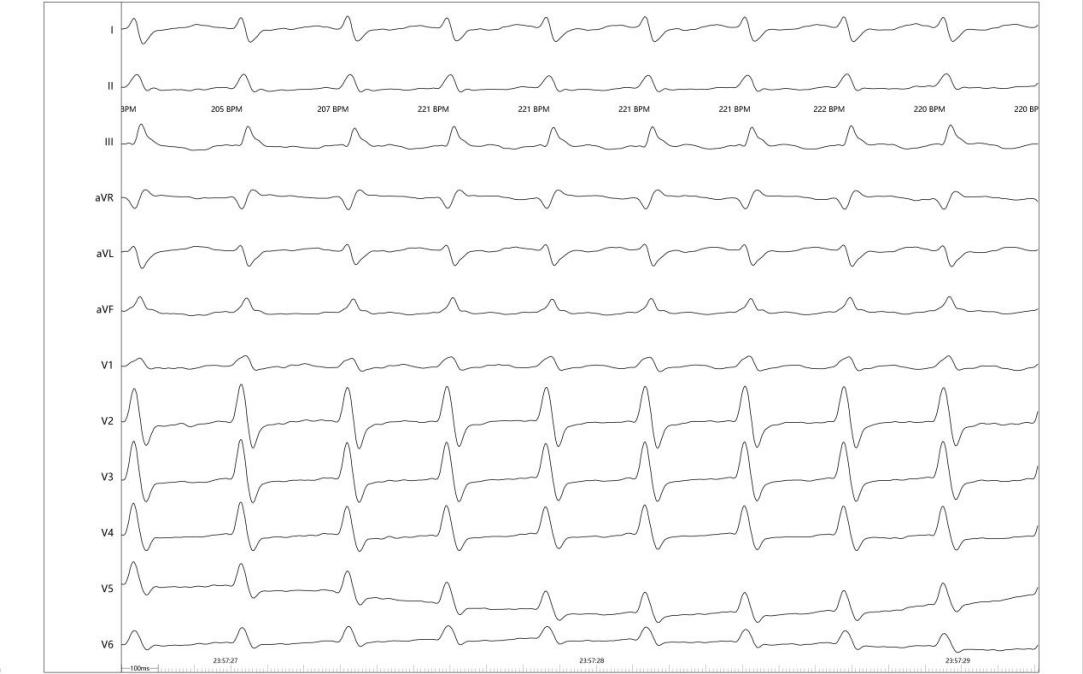


First-type atrial flutter


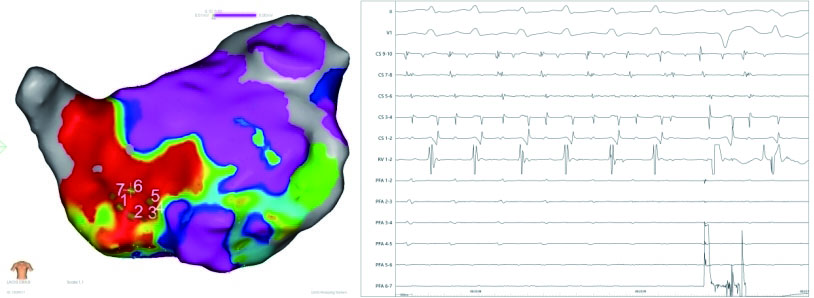


Left atrium (LA) bipolar map (0.1–0.5 mV) with right anterior wall and septal low-voltage areas. The picture on the right shows the potential of the low-voltage areas.


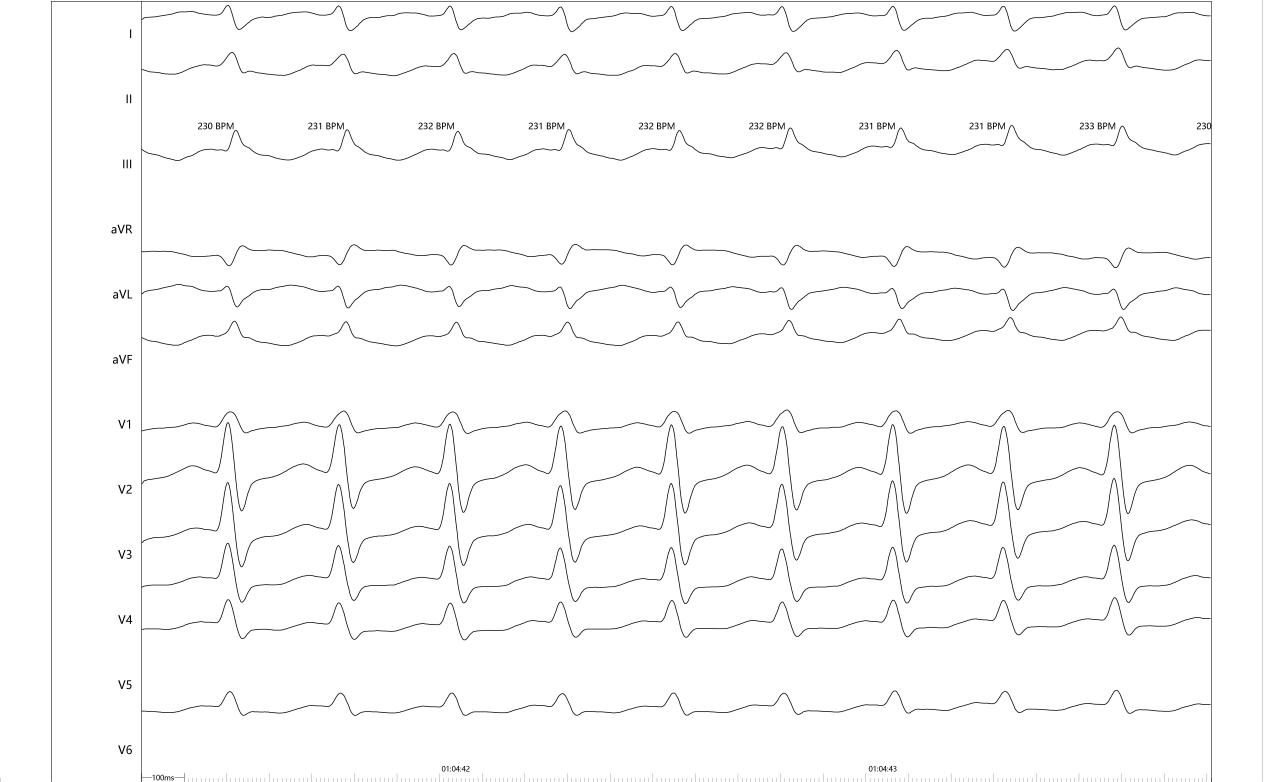


Second-type atrial flutter
